# Rapid Transformation in Wetting Properties of PTFE Membrane Using Plasma Treatment

**DOI:** 10.3390/polym15193874

**Published:** 2023-09-24

**Authors:** Shakila Parveen Asrafali, Thirukumaran Periyasamy, Seong-Cheol Kim

**Affiliations:** 1 School of Chemical Engineering, Yeungnam University, Gyeongsan 38541, Republic of Korea; shakilaparveen@yu.ac.kr; 2 Department of Fiber System Engineering, Yeungnam University, Gyeongsan 38541, Republic of Korea; thirukumaran@ynu.ac.kr

**Keywords:** PTFE, plasma treatment, active gas, N_2_ gas, surface modification

## Abstract

In this paper, we describe the surface modification of polytetrafluoroethylene (PTFE) through the plasma treatment process. Several parameters including different active gases, RF power, distance between the plasma source and sample, and plasma duration were optimized to reduce the hydrophobic nature of PTFE. Three different active gases were used (i.e., N_2_, O_2_, and (Ar+H_2_)); N_2_ was effective to reduce the hydrophobicity of PTFE within a shorter plasma duration of 2 min. Several surface characterizations including ATR-FTIR, water contact angle, FE-SEM, and XPS were utilized to verify the neat and modified PTFE surface after plasma treatment. The plasma treatment using N_2_ as an active gas improved the wettability of the PTFE membrane, showing a water contact angle of 109.5° when compared with the neat PTFE (141.9°). The SEM images of plasma-treated PTFE showed greater modifications on the surface indicating non-uniform fiber alignment and torn fibers at several places. The obtained results confirm the fact that plasma treatment is an effective way to modify the PTFE surface without altering its bulk property.

## 1. Introduction

Polytetrafluoroethylene (PTFE) is a fluoropolymer, with a molecular structure of (-CF_2_-CF_2_-)_n_. It is a registered trademark product of DuPont and commercially named ‘Teflon’. PTFE possess a hydrophobic surface with a surface free energy of 18.6 mJ/m^2^. This hydrophobic property is the outcome of low polarizability of C-F bonds in it. Due to this distinguishable hydrophobicity, PTFE can withstand harsh thermal, mechanical, and chemical conditions, possessing excellent thermal stability with increased working temperature range, wear resistance, low friction, and chemical inertness. These innumerable properties of PTFE make its usage wide in many fields including electronics (automobiles, semiconductors); medicinal (pharmaceuticals, medical instruments); household items (non-stick cookware); and environmental protection (paints, filtration membrane) [1,2,3,4,5,6,7,8,9,10,11]. Although the hydrophobic property of PTFE is admirable, due to this property, PTFE surfaces do not adhere to other material’s surface and moreover, it has high static voltage (5 kV~25 kV) which reduces its lifetime, particularly in the electronics field.

PTFE is one of the widely used low-cost hydrophobic materials for electro-wetting on dielectric applications. It is due to the hydrophobic and porous nature of this material which results in electrical breakdown. And therefore, a method to increase the adhesion of PTFE is necessary [7,8,9]. To broaden its application in various fields and to increase its lifetime, while maintaining its unique properties, surface modification of PTFE has been the focus of research in recent years. Surface modification of Teflon can be performed by both physical [12,13,14] and chemical methods [15,16]. However, physical methods (such as plasma treatment [17,18,19], sputtering [20,21], electron beam treatment [22], radiation grafting [23], laser treatment [24], atmospheric pressure plasma jet [25], particle beam irradiation, and irradiation of synchrotron radiation [26]) are generally adopted due to their high efficiency and because they do not cause any damage to the PTFE membrane. A balance between surface wettability and adhesiveness between the liquid (water) and solid surface (Teflon) is of great importance to produce hydrophilic and hydrophobic surfaces for various applications. In the case of chemical treatment methods, substitution of the fluorine atom by hydrophilic groups (such as hydroxyl and carbonyl groups) is very difficult, as the C-F bond has a high bond energy of 486 kJ/mole. And so, physical methods, in particular plasma treatment, are effective in producing hydrophilic PTFE surfaces.

Surface modification by plasma treatment is an inexpensive, clean, and scalable approach as it can induce a specific surface change without altering the bulk property of the material. Plasma treatment modifies the surface through physical bombardment by energetic ions, chemical reactions at the surface, and cross-linking. Even a short period of plasma treatment (a few seconds to a maximum of 1 min) is sufficient to bring about changes in surface wettability [9,12,27,28,29,30,31,32]. By optimizing the operating parameters (such as pressure, power, time, and gas flow rate) and by varying the plasma gas (such as Ar, N_2_, O_2_, Ar+H_2_, CO_2_, and NH_3_) different type of functionalization will be imparted on the surface of the material [33,34]. Liu et al. [35] studied the effects of dielectric barrier discharge (DBD) plasma surface treatment on three different polymer surfaces, viz., polytetrafluoroethylene (PTFE), polyimide (PI), and poly(lactic acid) (PLA). The influence of different operating parameters, including plasma power, plasma duration, and electrode gap were analyzed and it was found that plasma duration plays a significant role for PTFE and PI, whereas electrode gap plays a significant role for PLA. All the polymers showed improvement in wettability for all the parameters indicating appreciable changes (change in wettability, either hydrophobic to hydrophilic or vice-versa, inclusion of the respective functional groups or atoms) in their surface chemistry.

Sprik et al. [27] used argon plasma treatment to increase the wettability and photocatalytic activity of the hydrophobic polymer, FS-5 Dodec (a copolymer of dibenzo (b,d) thiophene sulfone monomer and di-n-dodecyl-9H-fluorene). The work showed that plasma treatment time of 20 min increased the photocatalytic activity about eight-fold, when compared with the untreated one. Pegalajar-Jurado et al. [33] enhanced the therapeutic activity of biopolymer by modifying the surface of hydrophobic polymer based on poly (lactic-co-glycolic acid) (PLGH) polymer. The S-nitrosated PLGH polymer after H_2_O plasma treatment becomes superhydrophilic, and still retains the S-nitrosothiol content for NO release. Guruvenket et al. [36] studied the plasma surface modification of polystyrene (PS) and polyethylene (PE). They used argon and oxygen plasma to modify the surface of the polymers with two different parameters, i.e., microwave power and treatment time. The study showed that an increase in microwave power increased the wettability of both the polymers, PS and PE, which were attributed to the surface functionalization and CASING (cross-linkage by activated species of inert gases). Bhowmik et al. [37] investigated the changes that occurred in wetting and physicochemical characteristics of polypropylene (PP) when it is exposed to a DC glow discharge (capacitively coupled discharge). The effect of DC glow discharge across three different electrodes including copper, nickel, and stainless steel on PP was studied in detail. The study revealed that stainless steel electrodes increased the surface energy and polar component of PP to a larger extent when compared with nickel and copper electrodes. In this work, an attempt has been made to decrease the hydrophobicity of PTFE using atmospheric plasma process. This method is simple and effective and costs less than the methods reported in previous works. We believe that different gases (N_2_, O_2_ and (Ar+H_2_)) used in the plasma process bring significant changes to the surface of PTFE. The changes that occurred in the sample were monitored by several characterization techniques and discussed in detail.

## 2. Materials and Methods

The PTFE sheet roll (thickness = 0.08 mm and length = 20 cm) was purchased from Flontec Co., Ltd., Incheon, Republic of Korea and different gases including Ar, N_2_, O_2_ and Ar+H_2_ (4%) were supplied from Korea standard gas (KSG), Gyeongsan, Republic of Korea.

### 2.1. Instrumentation Methods

An attenuated total reflectance Fourier transform infrared spectrometer ATR FT-IR, MB3000, from Perkin Elmer was employed to acquire the FT-IR spectrum. The infrared spectrum was obtained in the range 400–4000 cm^−1^. The neat PTFE and plasma treated PTFE were placed on the sample disc and the spectrum was recorded. For each sample, the spectrum was recorded at three different spots on the PTFE to observe the uniformity in the sample. In order to obtain high-resolution XPS spectra, K-Alpha (Thermo Scientific) was utilized. CasaXPS software software version number (2.3.22PR1.0) was used for deconvolution of high-resolution XPS spectra. A high-resolution scanning electron microscope (FESEM, Hitachi S-4800) at an accelerating voltage of 10 kV was employed to examine the morphology of the neat PTFE and plasma-modified PTFE. The working distance was about 8–9 mm and the SEM images were taken at different magnifications from 1 to 10 k. The samples were coated with platinum before analysis. A Dataphysics Instrument OCA 20 model was used to determine the water contact angle of the neat PTFE and modified PTFE. The measuring range of the instrument was from 0 to 180°. For each sample, contact angle measurement was taken in five different areas and the average value was denoted. 

### 2.2. Plasma Treatment Procedure

**Figure 1:**
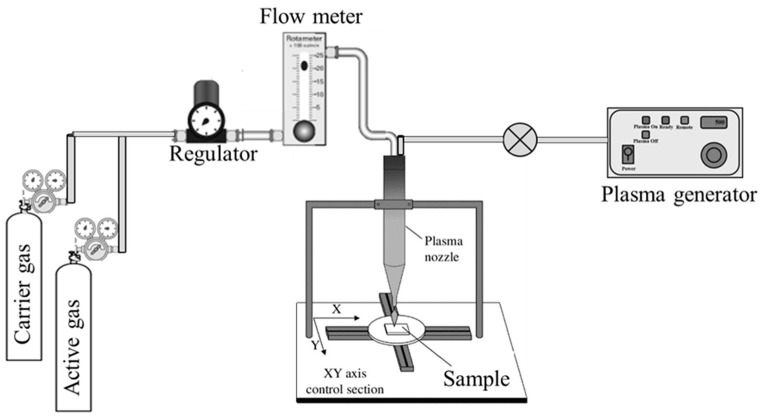
Schematic representation of atmospheric plasma treatment instrument.

The schematic representation of atmospheric plasma instrument is given in Figure 1. It consists of a sample holder, plasma generator, plasma nozzle, and flow meter. The sample holder is flat and large enough to place the sample in the horizontal direction. It can be moved back and forth horizontally up to a specific distance. The plasma generator acts as a source of plasma, whose intensity is adjusted using RF power (radio frequency) and passes through the plasma nozzle to the surface of the sample. The flow rate of the carrier gas and active gas is controlled by the flow meter. Generally, argon gas is used as a carrier gas and N_2_, O_2_, H_2_, or a mixture of gases is used as an active gas. There is a space provided just above the plasma nozzle, where the carrier gas, active gas, and plasma source are mixed together before depositing on the Teflon surface.

To start with, the Teflon sheet was cut into 15 × 15 cm and placed on the sample holder. A vacuum was applied to ensure proper attachment of the Teflon sheet to the sample holder. Care was taken to avoid wrinkles of the Teflon sheet. The sheet was placed as flat as possible without any wrinkles on it. Many parameters including the flow rate of the carrier and active gas, RF power, distance between the sample and plasma source, and plasma duration have been adopted and the details are given in the upcoming sections.

#### 2.2.1. Flow Rate of Carrier Gas

In the case of plasma treatment, Ar was used as the carrier gas. In general, the flow rate of the carrier gas should be much larger than the flow rate of active gas. Three different flow rates of carrier gas were used, i.e., 4.0 L/min, 4.5 L/min, and 5.0 L/min. It was found that 4.5 L/min was the optimum flow rate for all conditions. Increasing or decreasing the flow rate from 4.5 L/min increased the contact angle of water.

#### 2.2.2. Flow Rate of Active Gas

Different active gases (i.e., N_2_, O_2_ & (Ar+H_2_)) were chosen to modify the surface of PTFE and hence increase the wettability. These three different gases were chosen due to the hydrophilic nature of these gases. Once these gases are adsorbed on the surface of PTFE, they can abstract hydrogen and can produce several hydrophilic groups on the surface of PTFE. In general terms, the flow rate of the active gas should be much less than the carrier gas. Five different flow rates of these active gases were chosen (i.e., 10, 15, 20, 25, and 30 mL/min). Other than the 20 mL/min flow rate, all the other flow rates showed increased wettability, when checked using a syringe. And so, 20 mL/min was chosen as the optimum flow rate for the active gas.

#### 2.2.3. RF Power

The plasma power/RF power also plays an important role in producing changes on the surface of PTFE. The RF power was varied between 100 and 200 W (i.e., 100, 125, 150, 175, and 200 W). It was found that 150 W was appropriate, and increasing the RF power to 175 and 200 W, led to a discontinuous plasma source.

#### 2.2.4. Distance

A square-shaped sample holder (15 × 15 cm) was used to place the PTFE sheet and a rectangular plasma nozzle (15 × 2 cm) was used to deposit the plasma onto the PTFE sheet. The plasma nozzle could be moved in a horizontal direction, so that the plasma could be deposited all over the sample (i.e., PTFE sheet). The distance between the sample holder and the plasma nozzle could be varied between 1 and 3 mm (i.e., 1, 2, and 3 mm). It was found that a distance of 3 mm was appropriate. Both 1 mm and 2 mm distances caused rolling of the PTFE sheet along with the plasma nozzle and so the plasma treatment was not effective in both the conditions.

#### 2.2.5. Duration of Plasma Treatment

Duration of plasma treatment can be varied by changing the speed and repetition time. The speed and repetition time was set so that the plasma duration was varied between 1 and 4 min (i.e., 1, 2, 3, and 4 min). These four time durations were followed for all the active gases (i.e., N_2_, O_2_ & (Ar+H_2_)) and the differences in surface modification were analyzed. For the N_2_ plasma treatment, the following parameters were used:

Flow rate of Ar: 4.5 L/min;
Flow rate of N_2_: 20 mL/min;
RF power: 150 W;
Distance between the plasma nozzle and Teflon sheet: 3 mm;
Duration of plasma treatment: 1, 2, 3, and 4 min.

A pinkish glow of plasma could be seen with naked eyes when using N_2_ as an active gas. Similar parameters were followed for the O_2_ and Ar+H_2_ (4%) gases. A white glow was seen for the O_2_ plasma and a pale pink glow was seen for the Ar+H_2_ (4%) plasma. Table 1 shows the details of the different parameters used for the different active gases (i.e., N_2_, O_2_ and Ar+H_2_). Soon after plasma treatment, the PTFE sheet was carefully removed from the sample holder and packed in a zip-lock cover, to avoid its exposure to atmospheric gas. The plasma-treated side of the PTFE sheet was marked and several analyses were performed.

## 3. Results and Discussion

The plasma process was carried by fixing some parameters (volume flow rate of the carrier and active gasses, plasma/RF power, distance of the plasma nozzle) and varying other parameters (plasma duration and active gasses). A plasma duration of 1–4 min was adopted, considering that plasma treatment can bring about surface modifications within a few minutes and prolonged plasma exposure can cause degradation to the PTFE sheet (color change of the Teflon sheet was observed through naked eyes) [12]. For all the conditions of plasma treatment, the hydrophobic nature of neat Teflon was decreased, due to the incorporation of nitrogen and oxygen atoms.

### 3.1. FT-IR Analysis

**Figure 2:**
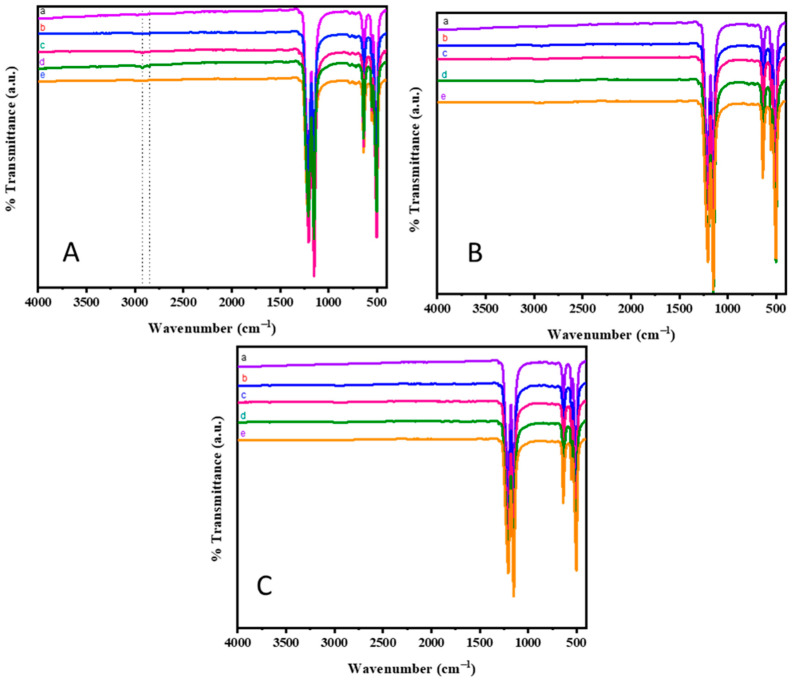
FTIR spectra of plasma treated samples (**A**) N_2_ plasma treatment; (**B**) O_2_ plasma treatment; and (**C**) Ar+H_2_ plasma treatment (a—neat teflon; b—1 min; c—2 min; d—3 min; and e—4 min).

**Figure 3:**
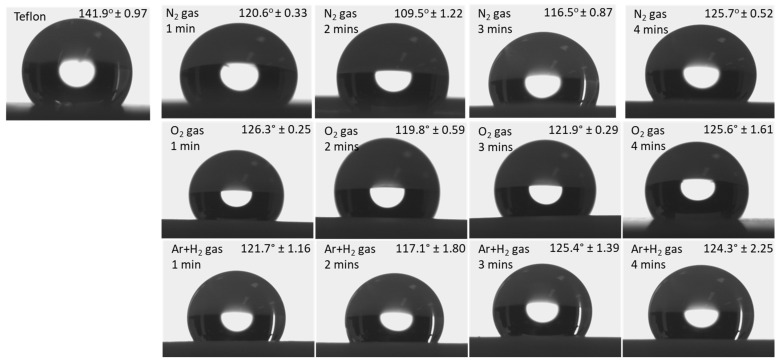
WCA images of untreated Teflon and plasma-treated Teflon with different gases.

**Figure 4:**
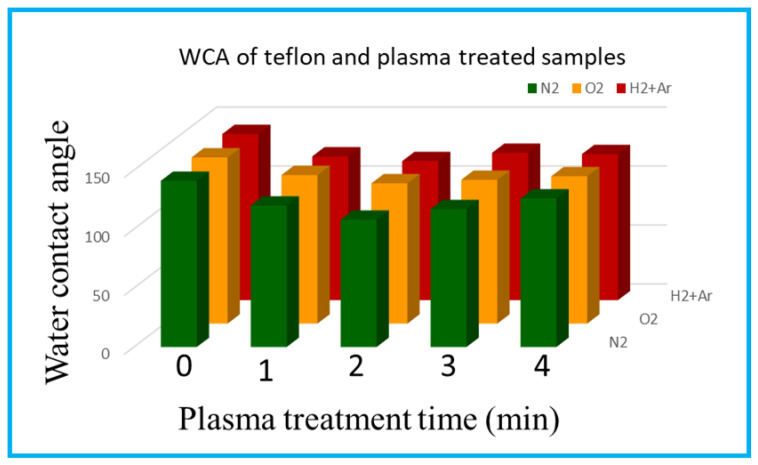
WCA images of untreated Teflon and plasma-treated samples.

The surface modification of Teflon by plasma treatment, using N_2_, O_2_, and (Ar+H_2_) as active gases was analyzed by FT-IR spectroscopy and is represented in Figure 2A–C. As evidenced, the neat PTFE sheet (Figure 2A(a)) showed an absorption band at 1205 and 1149 cm^−1^, due to the asymmetric and symmetric stretching vibrations of the C-F bond, respectively. In addition to this, the bands at 638, 525, and 501 cm^−1^ corresponded to CF_2_ rocking, CF_2_ deformation, and CF_2_ wagging modes, respectively [38,39]. The N_2_ plasma treatment for 2 min (Figure 2A(c)) and 3 min (Figure 2A(d)) showed a slight modification in the absorption spectrum. In addition to the CF_2_ bands, peaks with less intensity appeared at 2918 and 2845 cm^−1^, respectively, which corresponded to the stretching vibrations of the –CH or C-NH_2_ bond (Figure 2A(c,d)). The source of nitrogen was from the plasma treatment and it appeared as and -NH_2_ group [12]. Whereas the N_2_ plasma duration of 1 min (Figure 2A(b)) and 4 min (Figure 2A(e)) did not show any modifications with respect to the neat Teflon. Moreover, the FT-IR spectra of the O_2_ and (Ar+H_2_) plasma treatment (Figure 2B,C) did not show any visible changes in addition to the CF_2_ peaks. It could be visualized that the plasma treatment brought about very minute modifications on the PTFE surface that was not observed in the FT-IR spectrum. Even though the N_2_ and O_2_ plasma treatments tended to form C-N, C-O, and C-H bonds on the surface of Teflon, only changes with respect to the C-H bond could be observed in the FT-IR spectrum. Interestingly, the N_2_ plasma treatment for 3 min show the appearance of C-H stretching vibrations [12,40].

### 3.2. Water Contact Angle Analysis

The contact angle measurements were taken immediately to minimize any changes in the surface of the PTFE. A small part of the neat PTFE membrane and plasma-treated PTFE membrane (about 3 × 1 cm) was placed on the glass slide. Proper precaution was taken to place the PTFE membrane without folding it. A high-resolution camera with 6× zoom lens was used to capture the contact angle formed by the water droplet on the surface of the Teflon membrane. A micro-syringe containing DI water was used and the contact angle measurements were taken by placing 2 µL of DI water onto the membrane surface.

Figure 3 shows the static water contact angle measurements of neat PTFE and N_2_, O_2_, and (Ar+H_2_) plasma-treated PTFE as a function of the plasma exposure time. The neat PTFE membrane shows a contact angle of 141.9°, representing a very high hydrophobic surface (Figure 3). Whereas the modified PTFE membrane with the N_2_ plasma treatment shows a reduced contact angle, regardless of the plasma exposure times. Interestingly, N_2_ plasma exposure for 2 min reduces the hydrophobic nature of the PTFE membrane from 141.9° to 109.5°. Moreover, the increased plasma treatment of 3 and 4 min did not show any positive effect on the PTFE surface. Therefore, N_2_ plasma exposure for 2 min is sufficient to bring changes on the PTFE surface. And increased plasma exposure could damage the material’s surface and does not show any improvement. In spite of this, the other two plasma treatments (i.e., O_2_ and (Ar+H_2_)) reduce the contact angle only to a lesser extent up to 119.8 and 117.1°, respectively. Similar to the N_2_ plasma treatment, even for the O_2_ and (Ar+H_2_) plasma treatment, increasing the plasma exposure times, such as for 3 and 4 min, does not reduce the contact angle further and 2 min seems to be effective [27,39,40,41]. Prolonged plasma duration (more than 2 min) creates a damaged surface for all the plasma treatments (N_2_, O_2_, and Ar+H_2_), which further increases their hydrophobicity. Hence, among the three plasma treatment conditions, the N_2_ plasma seems to be effective even with the very less plasma exposure time of 2 min [42] (Figure 4).

### 3.3. SEM Analysis

**Figure 5:**
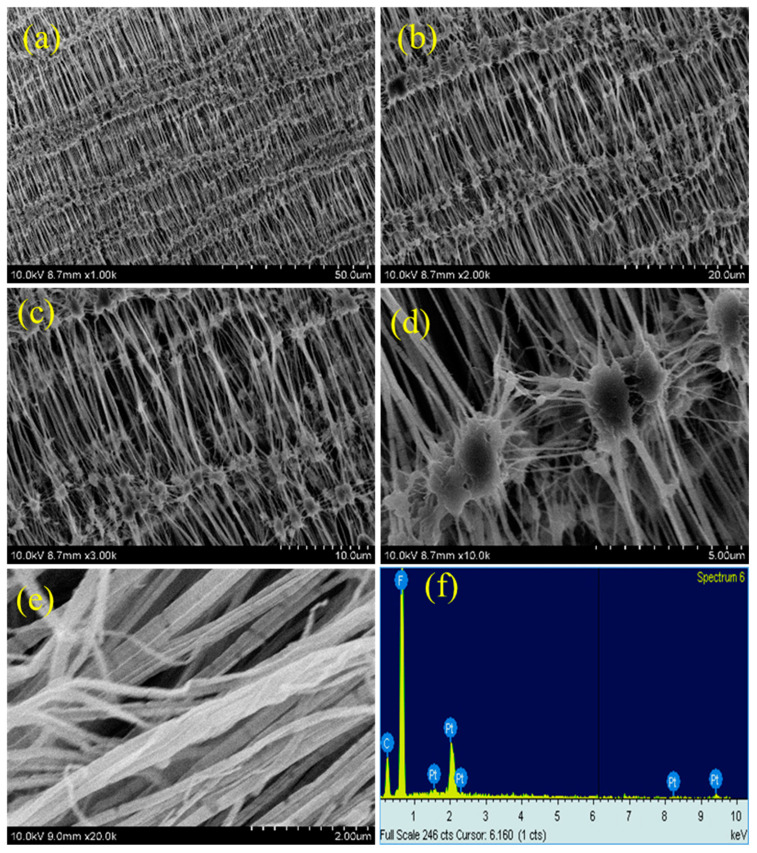
SEM images of untreated Teflon at different magnifications (**a**–**e**) with EDX (**f**).

**Figure 6:**
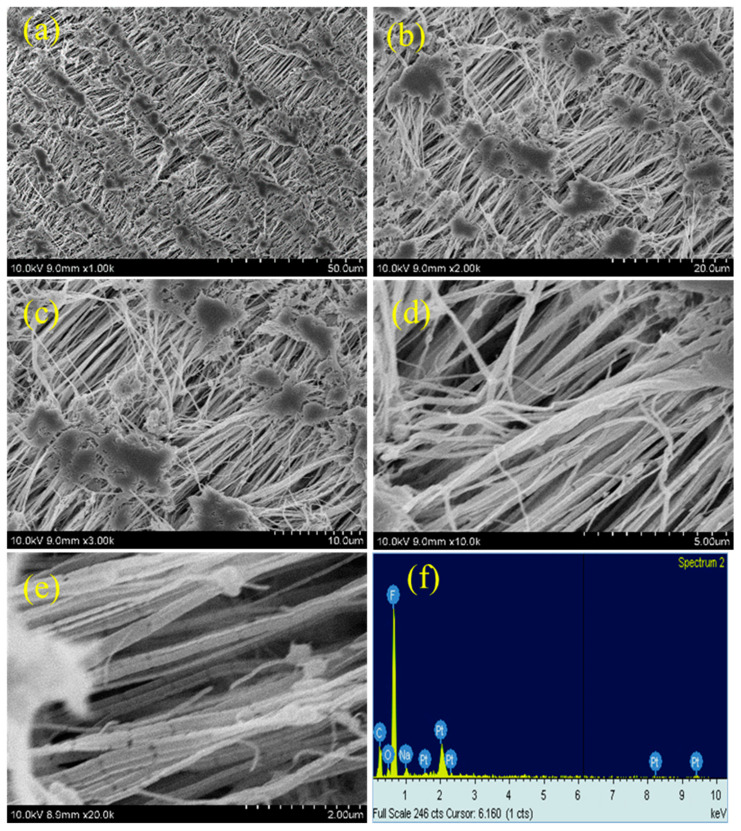
SEM images of N_2_ plasma-treated samples at different magnifications (**a**–**e**) with EDX (**f**).

**Figure 7:**
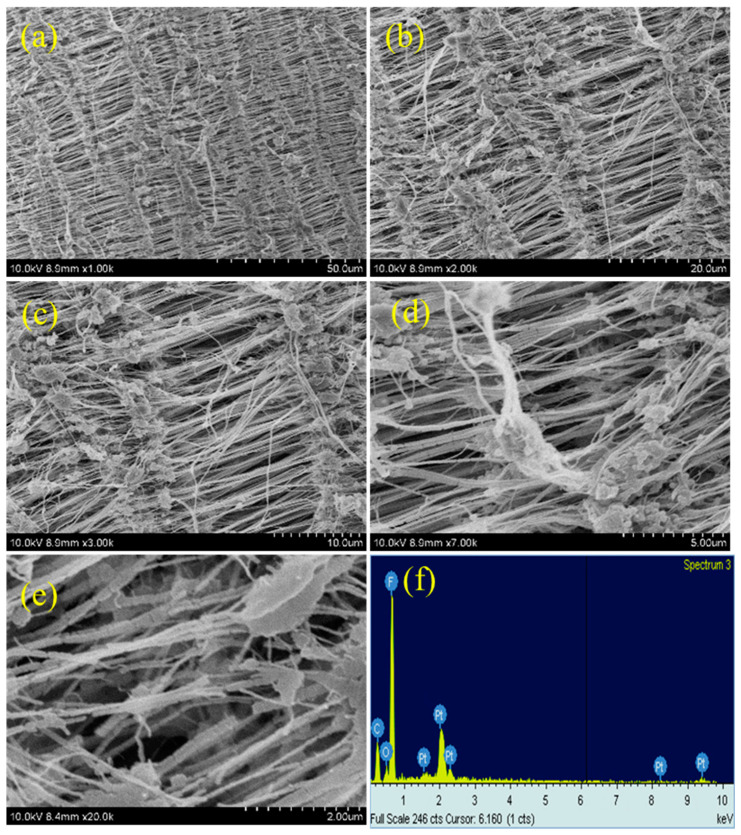
SEM images of O_2_ plasma-treated samples at different magnifications (**a**–**e**) with EDX (**f**).

**Figure 8:**
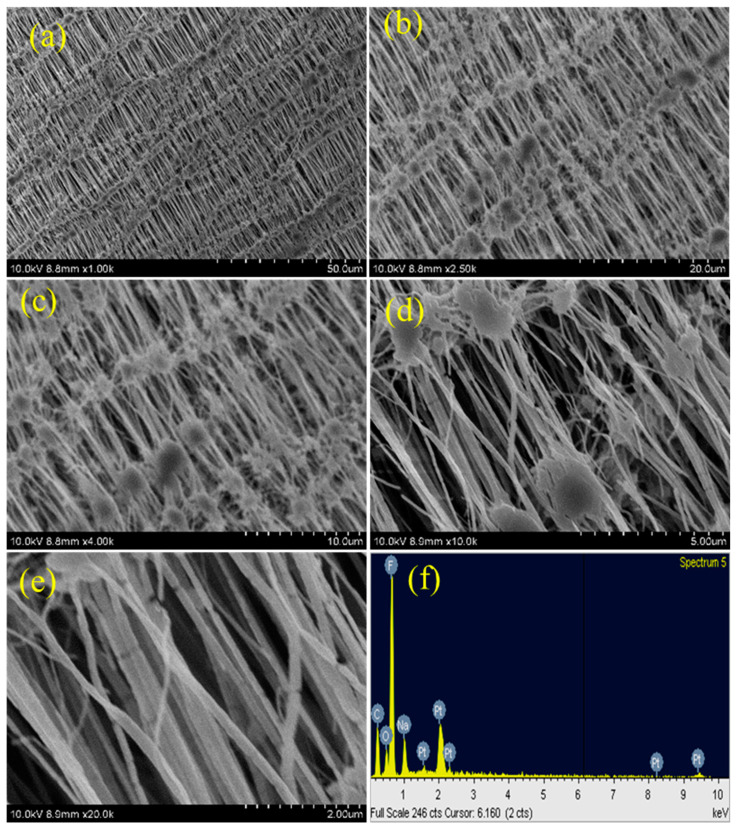
SEM images of Ar + N_2_ plasma-treated samples at different magnifications (**a**–**e**) with EDX (**f**).

As plasma treatment is a surface modification process, the surface morphology of Teflon is altered after the plasma treatment process. The morphology changes that occurred on the PTFE membrane after the different plasma treatments was investigated by FE-SEM measurements. Figure 5, Figure 6, Figure 7 and Figure 8 depict the SEM images of neat Teflon and the modified Teflon membrane after the plasma treatment process with N_2_, O_2_, and (Ar+H_2_) at different magnifications. As with the three plasma treatments (i.e., N_2_, O_2_ and (Ar+H_2_)), the water contact angle was much reduced with the 2 min exposure of plasma, and so the SEM images of Teflon with different plasma treatments for 2 min were discussed to figure out the maximum changes in the surface morphology. If we take a closer look at the SEM images, particularly at higher magnifications, the surface morphological changes can be clearly seen.

The neat PTFE membrane (Figure 5a–f) shows several reticular fiber-like nodule structures, in which the fibers are aligned in a uniform order and connected to another fiber through this nodule (Figure 5c). Even at a higher magnification, the SEM images show uniform alignment of the fibers, representing bundles of rods stacked together. It can also be observed that from each nodule several fibers arise that are connected together (Figure 5d). 

This reticular fiber–nodule structure is a result of melt-stretching membrane fabrication technology adopted to fabricate the Teflon membrane, and the hydrophobic nature of the Teflon membrane is expected to be from this structure contribution in addition to the –CF_2_ groups. For the plasma-treated PTFE membrane, low magnification SEM images, e.g., 1k, do not show many significant changes (Figure 6a, Figure 7a and Figure 8a), whereas, the high magnification SEM images, show drastic changes on the surface morphology.

**Figure 9:**
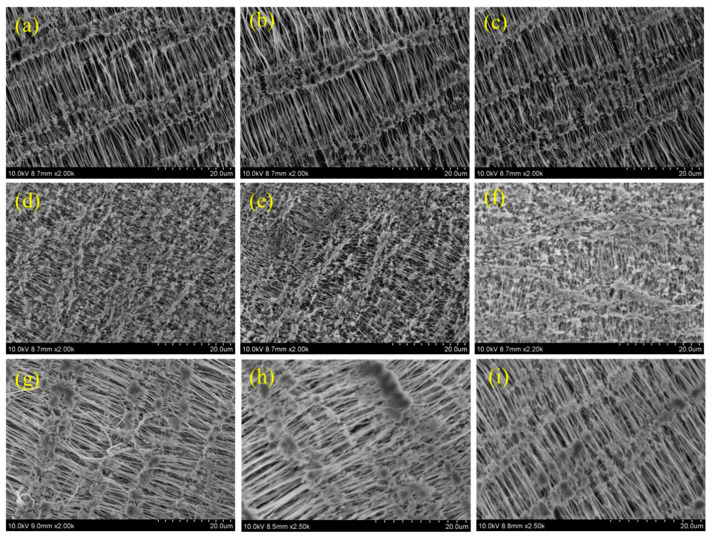
SEM images of different plasma-treated samples (**a**–**c**) N_2_ at 1, 3, and 4 min; (**d**–**f**) O_2_ at 1, 3, and 4 min; and (**g**–**i**) Ar+H_2_ at 1, 3, and 4 min.

At first, the alignment of the fiber structure is completely collapsed, and at several places the fibers are torn and broken fibers are clearly visible (Figure 6b–e, Figure 7b–e and Figure 8b–e) [39,40]. And so, we can confirm that all the plasma treatment brings drastic changes with reference to the surface morphology of the Teflon membrane. Even though the O_2_ and (Ar+H_2_) plasma treatment modify the surface morphology of Teflon similar to the N_2_ plasma treatment, they do not reduce the contact angle much. This could be attributed to the fact that soon after the plasma treatment, the atmospheric air can further affect the surface of the Teflon membrane. The atmospheric oxygen could be deposited on the Teflon membrane and with the N_2_ plasma, it is less pronounced. This could be the reason that the N_2_ plasma treatment is more effective than the O_2_ and (Ar+H_2_) plasma treatment. The EDX spectrum (Figure 5f, Figure 6f, Figure 7f and Figure 8f) gives the composition of each element present in the Teflon membrane. It can be observed that ‘C’ and ‘F’ are the predominant elements present in all samples. The neat Teflon membrane shows the presence of a very little amount of ‘O’ species, whereas all the plasma-treated samples show an increase in the percentage of oxygen. The SEM images of the other samples, N_2_, O_2_, and Ar+H_2_ at 1, 3, and 4 min of plasma duration is shown in Figure 9. As can be seen, the morphology of PTFE is not affected with a plasma duration below or above 2 min.

### 3.4. XPS Analysis

**Figure 10:**
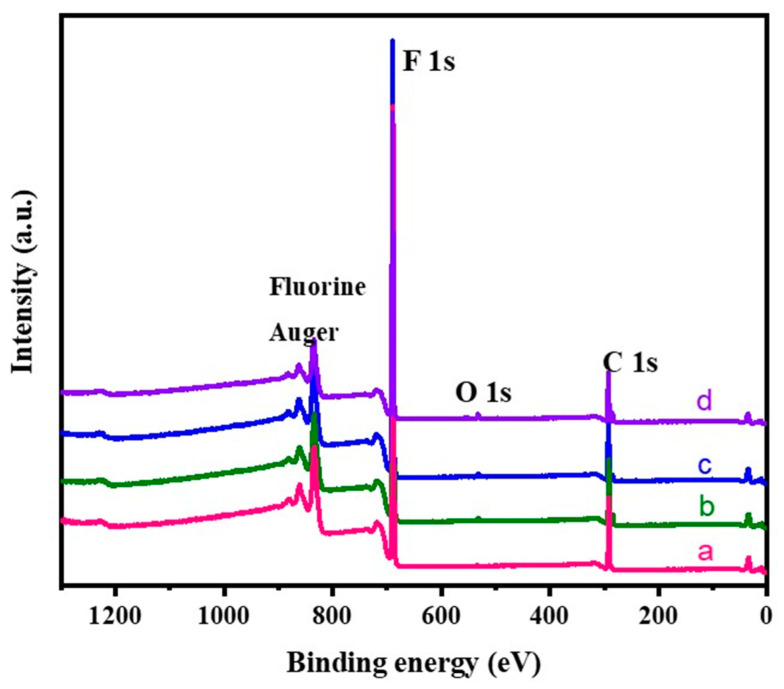
XPS survey spectra of untreated Teflon (a); plasma-treated samples with N_2_ (b); O_2_ (c); and Ar+H_2_ (d).

The atomic composition present on the surface of the neat PTFE sheet and plasma-treated PTFE sheets with N_2_, O_2_, and (Ar+H_2_) gases were determined by XPS analysis. Figure 10 represents the XPS survey spectra of the neat PTFE sheet and plasma-treated PTFE sheets. From the survey spectrum, it was clearly observed that ‘C’ and ‘F’ were the predominant species found in all the samples. The neat PTFE showed peaks for C1s at 294 eV, and for F1s at 683 eV (Figure 10a). Whereas for the plasma treated samples, an additional small peak at 532 eV was obtained, corresponding to O1s binding energy (Figure 10b–d). This clearly indicated that the surface of the PTFE had been modified by the plasma treatment. The data in Table 2 provides an indication about the atomic percentage of different elements present on the PTFE surface. It is observed from the data that there is an increase in the percentage of carbon and a decrease in the percentage of fluorine for all the plasma treatments. Additionally, a much lower percentage of ‘N’ and ‘O’ atoms were also observed for the plasma-treated samples in comparison with the neat PTFE. The data clearly shows that all the plasma treatments (i.e., N_2_, O_2_, and (Ar+H_2_)) brought about defluorination of the PTFE, as a result of C-F chain breaking in the polymer induced by the electrons generated in the plasma. And hence, we can observe a decrease in the fluorine content when compared with the neat PTFE. Moreover, all the plasma treatments results with an introduction of ‘N’ and ‘O’ atoms. This is attributed to two factors, viz., the active gas used in the plasma treatment and the surface oxidation of the PTFE sheet by atmospheric air soon after the plasma treatment. In all the cases, a very short plasma duration of 2 min was sufficient to bring out these changes [9,12,27].

**Figure 11:**
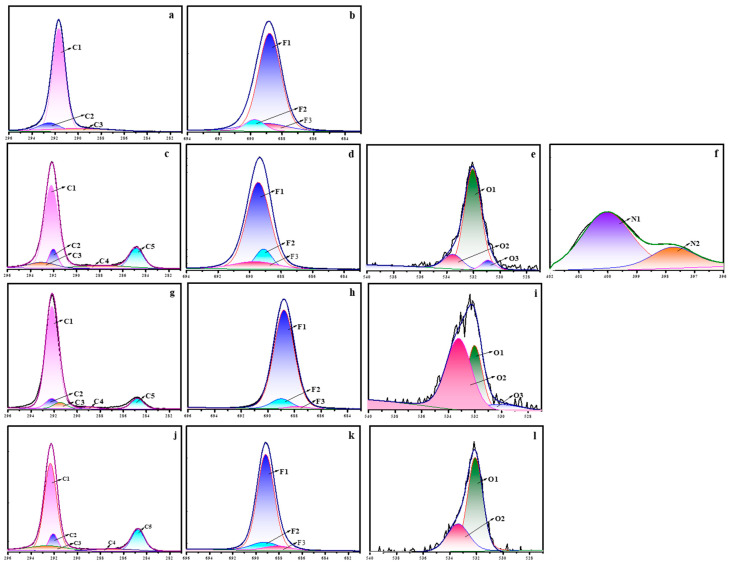
XPS deconvolution spectra of untreated Teflon (**a**,**b**); plasma-treated samples with N_2_ (**c**–**f**); O_2_ (**g**–**i**); and Ar+H_2_ (**j**–**l**) (*X*-axis: Binding energy (eV), *Y*-axis: Counts/s (a.u.)).

The high-resolution XPS spectra showing the binding energy of C1s, F1s, N1s, and O1s are depicted in Figure 11. The C1s spectrum of neat PTFE was deconvoluted into three different peaks, representing three different chemically non-equivalent carbon atoms (Figure 11a). The major peak at 291.8 eV was related to the CF_2_ species and the two smaller peaks at 292.3 and 288.6 eV were related to the CF_3_ and CF/C-C species, respectively. Similarly, the high resolution F1s spectra of neat PTFE (Figure 11b) showed three different peaks. The one with higher intensity at 688.7 eV corresponded to the F-C-F species and the other two with very small intensities at 689.7 and 689.0 eV corresponded to the F_3_-C and F-C species, respectively. For all the plasma treated samples, the C1s spectra showed very small additional peaks at 285 and 288 eV, representing the presence of C-O and C-H species (Figure 11c,g,j). Additionally, there was a decrease in the CF_2_ peaks for all the plasma-treated samples, which was more prominent for the N_2_ plasma treatment (Figure 11c). In the same way, the F1s spectra also showed a decrease in the F-C-F peaks (Figure 11d,h,k). In addition to C1s and F1s, the N_2_ plasma treatment showed additional peaks due to O1s and N1s (Figure 11e,f), whereas the O_2_ and (Ar+H_2_) plasma treatment showed additional peaks due to O1s (Figure 10i,l). The O1s spectra was deconvoluted into three different peaks corresponding to C-O, C=O and C-OH species and the N1s spectra was deconvoluted into two different peaks, representing the C-N and N-H species [39,40]. And therefore, the combined results of FT-IR, SEM, WCA, and XPS indicates that plasma treatment brings about surface modification of neat PTFE membrane.

## 4. Conclusions

The work reported in this paper demonstrates a simple and effective method to modify the surface of PTFE without altering its bulk property by tuning the hydrophobic nature of PTFE. Plasma treatment was found to be successful in altering the surface morphology of PTFE by optimizing several parameters. The optimum parameters for plasma discharge were set as follows: flow rate of carrier gas (Ar) = 4.5 L/min; flow rate of active gas (N_2_/O_2_/(Ar+H_2_)) = 20 mL/min; RF power = 150 W; distance = 3 mm; and plasma exposure time = 1–4 min. The plasma treatment using N_2_ as an active gas was effective to reduce the hydrophobicity of PTFE from 140° to 108°, even with a very short duration time (2 min). In support of this result, the SEM images clearly indicated the modified PTFE surface after plasma treatment. The surface of PTFE was collapsed to a greater extent with uneven fiber orientation and broken fibers. Additionally, FT-IR confirmed the presence of –CH stretching vibrations and XPS confirmed the presence of oxygen and nitrogen atoms at 532 and 400 eV, respectively, in addition to carbon and fluorine atoms at 294 and 683 eV, respectively, (‘C’ and ‘F’ atoms are presumably found in neat PTFE). Moreover, the intensity of CF_2_ decreased with an increase in C-C/C-H intensity, which proved that plasma treatment results in defluorination with the formation of new polar groups appearing on the surface of PTFE. Overall, the results proved that the ability to tune the surface properties while maintaining the bulk property could be achieved effectively by plasma treatment. Even though plasma treatment could modify the surface of PTFE, the plasma treatment alone could not produce a hydrophilic surface and the produced hydrophilicity could not be retained for a longer period of time. This work is a preliminary study investigating the surface modification of PTFE by plasma treatment. To further enhance the surface modification and to maintain the modified surface, treatment with hydrophilic compounds before or after plasma treatment will be much more effective.

## Figures and Tables

**Table 1:** Different parameters used for plasma treatment.

Plasma Treatment	Carrier Gas		Active Gas		Distance (mm)	Duration (min)
	Gas	Flow Rate (L/min)	Gas	Flow Rate (mL/min)		
N_2_ Plasma treatment	Ar	4.5	N_2_	20	3	1–4
O_2_ Plasma treatment	Ar	4.5	O_2_	20	3	1–4
H_2_ Plasma treatment	Ar	4.5	Ar+H_2_ (4%)	20	3	1–4

**Table 2:** XPS data showing the atomic % of different elements.

Plasma Treatment	Atomic % of Different Elements			
	C	N	O	F
Teflon sheet	32.26	-	-	67.51
N_2_ plasma	34.06	0.48	1.81	63.39
O_2_ plasma	34.26	-	1.33	64.22
Ar+H_2_ plasma	34.79	-	1.23	63.79

## Data Availability

The data presented in this study are available in the article.
